# High-dose intravenous methylprednisolone therapy in patients with Graves’ orbitopathy is associated with the increased activity of factor VIII

**DOI:** 10.1007/s40618-018-0907-z

**Published:** 2018-06-09

**Authors:** P. Miśkiewicz, J. Milczarek-Banach, B. Rutkowska-Hinc, A. Kondracka, T. Bednarczuk

**Affiliations:** Department of Internal Medicine and Endocrinology, Medical University of Warsaw, Banacha 1a, 02-097 Warsaw, Poland

**Keywords:** Coagulation, Graves’ orbitopathy, Methylprednisolone, Pulmonary embolism, Venous thromboembolic event

## Abstract

**Purpose:**

Venous thromboembolic events (VTE), with their life-threatening manifestation as pulmonary embolism, are potential adverse effects of intravenous methylprednisolone (IVMP) pulse therapy, partially due to a hypercoagulable state. The aim of the study was to analyze the influence of IVMP on selected hemostatic parameters in patients with moderate-to-severe Graves’ orbitopathy (GO).

**Methods:**

26 euthyroid patients with GO were treated with 12 pulses of IVMP (6 × 0.5, 6 × 0.25 g every week). Hemostatic variables [factor (F) II, FV, FVII, FVIII, fibrinogen, antithrombin, activated partial thromboplastin time (aPTT), prothrombin time, international normalized ratio of prothrombin time, platelets and D-dimer] were analysed before, 24 and 48 h after 1st, 6th and 12th pulse.

**Results:**

A constant, transient trend in changes of some hemostatic variables was observed after all assessed pulses. We discovered an increase in median activity of FVIII 24 and 48 h after pulses, with a shortening of aPTT 24 h after each IVMP pulse (*p *< 0.00005). FVII decreased 24 h after each pulse (*p *< 0.0005 after 1st and 12th, *p *< 0.00005 after 6th). Fibrinogen level decreased 48 h after each pulse (*P *< 0.00005). We did not observe any statistically significant changes in hemostatic parameters in the long-term evaluation. Therapy was concluded in one patient after the 9th pulse due to pulmonary embolism.

**Conclusions:**

The increase of FVIII activity is a consequence of treatment with IVMP and occurs after each pulse. In patients with additional risk factors of VTE, anticoagulation prophylaxis should be considered.

**Electronic supplementary material:**

The online version of this article (10.1007/s40618-018-0907-z) contains supplementary material, which is available to authorized users.

## Introduction

The alterations of coagulation and fibrinolysis parameters have been described in patients with endogenous Cushing’s syndrome (CS) [1–5] and those treated with glucocorticosteroids (GCs) [6–8].This change in hemostatic process is associated with an increased risk of venous thromboembolic events (VTE) and pulmonary embolism (PE) [5, 9]. Anticoagulation prophylaxis reduces thromboembolic complications in endogenous and exogenous hypercortisolism [4, 10, 11]. The impact of the intravenous GCs therapy on hypercoagulability, however, remains unclear and perplexing. According to the European Group On Graves’ Orbitopathy (EUGOGO), patients with active, severely symptomatic and sight-threatening Graves’ orbitopathy (GO) should be treated with high dose intravenous methylprednisolone (IVMP) pulses [12, 13]. There are, however, reports of fatal side effects that may be associated with this therapy (e.g., PE, myocardial infarction, severe cerebrovascular events, acute liver damage and sudden death) [14–17]. For this reason, the cumulative dose of IVMP should not exceed 8 g within each treatment course, and pulses should not be given on consecutive or alternate days, except for the case of dysthyroid optic neuropathy [13, 15, 18]. Nevertheless, even smaller cumulative therapy may be associated with fatal cardiovascular complications [19]. Hence the aim of our study was to evaluate the effects of IVMP therapy on hemostatic process in patients with GO.

## Patients and methods

### Patients

This prospective study was conducted at an academic referral centre. Patients with active, moderate-to-severe GO according to EUGOGO classification were consecutively recruited in the Department of Endocrinology at the Medical University of Warsaw from 2011 to 2014. The inclusion criteria were: active, moderate-to-severe GO; age ≥ 18 years; euthyroidism for at least 1 month (patients with hyperthyroidism treated with anti-thyroid drugs, after radiotherapy/surgical treatment on levothyroxine therapy if necessary, with euthyroid GO, or with Hashimoto disease on levothyroxine therapy); completion of at least the first six IVMP pulses. Exclusion criteria were: medical history of thromboembolic events; cardiovascular morbidity (chronic heart failure, cardiovascular heart disease); uncontrolled hypertension (defined as systolic blood pressure ≥ 140 mmHg and/or diastolic blood pressure ≥ 90 mmHg); liver disease (defined as > 3 × increase of alanine aminotransferase and/or aspartate aminotransferase); active inflammation; nephritic syndrome; active neoplastic disease; previous GCs therapy within the last 6 months; trauma/surgery within the last 3 months; pregnancy or a bedridden state; use of: heparin, vitamin K antagonists, antiplatelet drugs, contraceptives or hormone replacement therapy. 26 patients were eligible for the study. All but two underwent the entire treatment schedule. Characteristics of the study group are shown in Table 1.

**Table 1:** Characteristics of patients (*n* = 26)

Characteristics	*n*	*%*
Female	15	58
Etiology of GO		
Graves’ disease	21	81
Hashimoto disease	5	19
Smoking		
Current	14	54
Former	8	31
Never	4	15
BMI ≥ 30 kg/m^2^	5	19
Comorbidities		
Hypertension	11	42
Diabetes	0	0
Hyperlipidemia	11	42
COPD	2	8
Crohn’s Disease	1	4
Medications		
Levothyroxine	18	69
Thyrostatics	12	46

	Median	Range
Age (years)	54	24–75
Duration of GO (weeks)	41	11–156
BMI (kg/m^2^)	25.1	19.3–34.8
Laboratory measurements		
TSH (normal range: 0.27–4.2 µIU/ml)	1.5	0–4.1
FT4 (normal range: 12–22 pmol/l)	16	12–22
CRP (normal range: 0–10 mg/l)	1.7	0.2–7.5

*BMI* body mass index, *COPD* chronic obstructive pulmonary disease, *CRP* C-reactive protein, *FT4* free thyroxine, *GO* Graves’ orbitopathy, *n* number of subjects, *TSH* thyroid stimulating hormone

### Study design

The participants received IVMP according to EUGOGO recommendations (cumulative dose of methylprednisolone 4.5 g, treatment duration 12 weeks in single weekly intravenous pulses, first 6 weeks 0.5 g of IVMP, next 6 weeks 0.25 g of IVMP). Hemostatic variables [factor (F) II, FV, FVII, FVIII, fibrinogen, antithrombin (AT), activated partial thromboplastin time (aPTT), prothrombin time (PT), international normalized ratio of prothrombin time (INR), platelet count (PLT) and D-dimer] were analysed before, 24 h (24 h) and 48 h (48 h) after selected (1st, 6th and 12th) IVMP pulses.

### Laboratory measurements

Venous blood was collected in the morning following a 12-h fast. This was utilized for preparation of serum, 3.2% sodium citrate plasma, and citrate platelet—poor plasma after double centrifugation.

Thyrotropin (TSH) and free thyroxine (FT4) were measured in serum using an electrochemiluminescence immunoassay (Roche Diagnostics, Mannheim, Germany) on Cobas 6000 Analyzer (Hitachi, Tokyo, Japan). D-dimer was determined by the immunoenzymatic method on analyzer VIDAS PC with VIDAS D-dimer Exclusion II (BioMérieux SA, Marcy l’Etoile, France). C-reactive protein (CRP) was measured in serum using an immunoturbidimetric method on Cobas 6000 Analyzer (Roche Diagnostics). PLT were determined by the impedance method on Sysmex XT Hematology Analyzer.

Hemostatic parameters were analyzed within 1–1.5 h after blood sampling, except for FVIII activity measured in platelet—poor plasma stored frozen at − 70 °C until assay.

Coagulation analyzer Option 4 (Biomerieux, Germany) was used for the measurement of aPTT with Dapttin TC (Technoclone GmbH, Vienna, Austria), PT with rabbit brain thromboplastin (Technoplastin HIS, Technoclone) and factors: II, V and VII activities in one stage method with factor—deficient plasma (Technoclone).

Analyzer BCS-XP (Siemens GmbH, Marburg, Germany) was used for fibrinogen measurement by Clauss method with Multifarben U, AT activity by the chromogenic method with Berichrom. AT and activity of FVIII by coagulometric method with FVIII—deficient plasma; all reagents from Siemens Healthcare Diagnostics Products GmbH (Marburg, Germany).

### Outcome analysis

The end point of the study was a change in hemostatic variables’ levels in laboratory tests. There were short- and long-term hemostatic changes analysed during IVMP therapy: comparisons of laboratory tests before, 24 and 48 h after selected pulses, and between the beginning of 1st, 6th and 12th IVMP pulses, respectively. Moreover, analyses were performed concerning clinical data (such as age, sex, body mass index, smoking, duration time of GO, presence of hypertension, basal markers of thyroid function) between independent groups (patients with initially increased/reduced selected markers versus without increased/reduced selected markers).

### Statistics

Statistical analysis was performed using STATISTICA software ver. 10.0. Continuous variables were demonstrated as mean ± standard deviation (SD) or median values (lower quartile–upper quartile). Categorical data was presented as numbers (*n*) or percentages (%). Changes of hemostatic variables between selected time points of the study were compared using parametric Student’s *t* test or non-parametric Wilcoxson matched pairs test. As far as analysis of clinical data between independent groups (patients with initially increased/reduced selected markers versus without increased/reduced selected markers) is concerned, the following tests were used: Chi-squared method for categorical variables or Mann–Whitney *U* test for continuous data. After Bonferroni correction, results with a *p* value of < 0.0005 were deemed statistically significant.

## Results

### Evaluation before intervention

Baseline median values of all parameters are shown in Table 2. FVIII activity at this point was higher than 150, 175 and 200% in 38, 23 and 4% of patients, respectively. Slightly increased activity of FII, FV, FVII, AT and D-dimer was observed in 4, 4, 15, 15 and 12% of patients, respectively. In all patients aPTT, PLT, and CRP remained within the reference range. We noticed a higher prevalence, without statistical significance, of increased basic FVIII activity in obese (BMI ≥ 30 kg/m^2^) than in non-obese patients (60 vs 33%, respectively).

**Table 2:** Changes in coagulation parameters during 1st intravenous methylprednisolone pulse (0.5 g)

Coagulation parameter	Before pulse	24 h after pulse	48 h after pulse
FII (Reference range 70–120%)	92.5 (84–105)	95.5 (90–108)	94 (82–104)
FV (Reference range 70–120%)	93.5 (78–109)	101.5 (91–117)^b^	89.5 (83–107)
FVII (Reference range 70–120%)	92 (81–112)	**80.5 (68**–**92)**^c^	86.5 (74–101)
FVIII (Reference range 70–150%)	140.2 (109.4–163)	**174.8 (150**–**247.8)**^d^	**166.5 (131**–**197.2)**^d^
PT (Reference range 12–16 s)	15.1 (14.5–16.4)	15.55 (15–16.7)^b^	15.7 (15.1–16.4)^a^
INR (Reference range < 1.3)	0.97 (0.93–1.05)	1.02 (0.97–1.09)^a^	1.01 (0.96–1.08)^a^
aPTT (Reference range 25–37 s)	31 (29–33)	**29 (27**–**30)**^d^	30 (27–32)^a^
Fibrinogen (Reference range 200–400 mg/dl)	321.5 (255–345)	312 (248–343)	**240 (207**–**282)**^d^
AT (Reference range 80–120%)	110.5 (104–115)	113.5 (108–119)^b^	110.5 (103.5–116)
D-dimer (Reference range < 500 ng/dl)	311 (226–395)	295 (225–416)	257 (189–383)
PLT (Reference range 150–400 × 10^3^/µl)	233 (190–266)	**258 (213**–**281)**^d^	229 (193–248)

Results are demonstrated as median values (lower quartile–upper quartile)

*FII* factor II, *FV* factor V, *FVII* factor VII, *FVIII* factor VIII, *PT* prothrombin time, *INR* international normalized ratio of prothrombin time, *aPTT* activated partial thromboplastin time, *AT* antithrombin, *PLT* platelets

After Bonferroni correction, results were claimed statistically significant with *p* value of < 0.0005 (bolded)

Statistical analysis was performed with paired *t* student test (parametric data) or with Wilcoxson test (non-parametric data)

^a^*p* < 0.05; ^b^*p* < 0.01; ^c^*p* < 0.0005; ^d^*p* < 0.00005, *p* values refer to comparisons with levels of coagulation parameters before the 1st pulse

### Short-term influence of single IVMP pulse on hemostasis (0.5 g of IVMP and 0.25 g of IVMP)

**Fig. 1:**
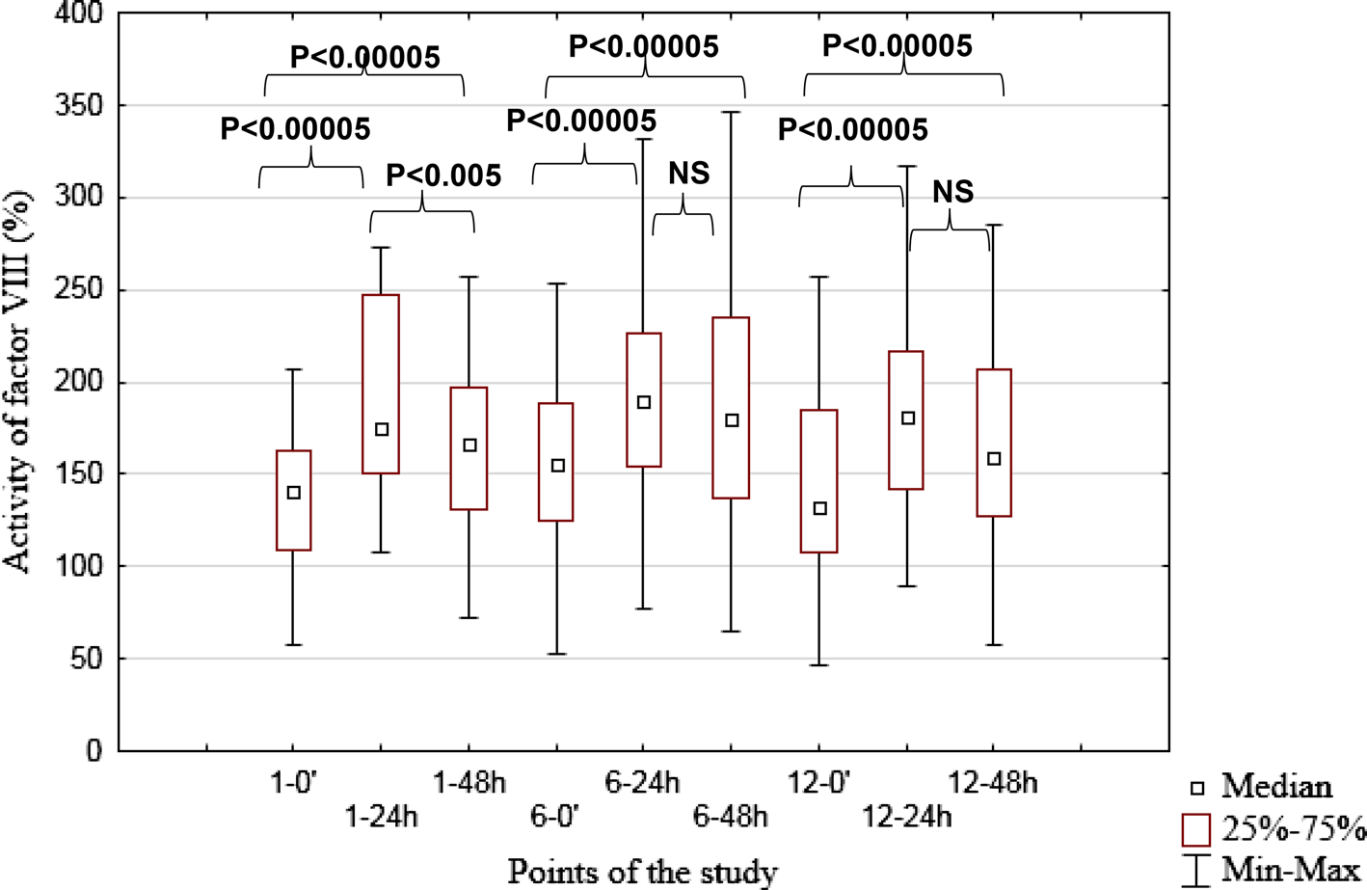
Activity of factor VIII during selected intravenous methylprednisolone pulses (normal range 70–150%).* Max* maximum,* Min* minimum,* NS* –not significant,* 1-0’* before 1st intravenous methylprednisolone pulse (IVMP),* 1–24h* 24 h after 1st IVMP,* 1–48h* 48 h after 1st IVMP,* 6-0’* before 6th IVMP,* 6–24 h* 24 h after 6th IVMP,* 6–48 h* 48 h after 6th IVMP,* 12-0’* before 12th IVMP,* 12–24 h* 24 h after 12th IVMP,* 12–48h* 48 h after 12th

**Fig. 2:**
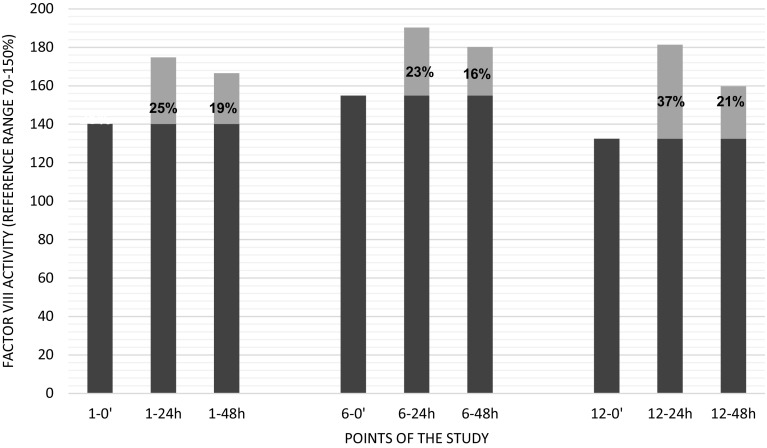
Increase of median factor VIII activity from the baseline to 24 and 48 h after selected intravenous methylprednisolone pulses.* IVMP* intravenous methylprednisolone pulse;* 1-0’* before 1st IVMP,* 1–24h* 24 h after 1st IVMP,* 1–48 h* 48 h after 1st IVMP,* 6-0’* before 6th IVMP,* 6–24 h* 24 h after 6th IVMP,* 6–48h* 48 h after 6th IVMP,* 12-0’* before 12th IVMP,* 12–24 h* 24 h after 12th IVMP,* 12–48 h* 48 h after 12th

Detailed outcomes of coagulation parameters for the 1st (0.5 g) IVMP pulse are shown in Table 2, and for the 6th (0.5 g) and 12th (0.25 g) IVMP pulses in Online Resources 1 and 2. The constant trend in changes of selected hemostatic variables was observed during all assessed pulses (1st, 6th and 12th). The following changes were noticed: increased activity of FVIII 24 h and 48 h after IVMP pulses (Figs. 1 and 2), decrease of aPTT 24 h after IVMP pulses, decrease of FVII 24 h after IVMP pulses, and decrease of fibrinogen 48 h after IVMP pulses. Some additional changes were observed in other parameters, but not at all points of evaluation (details in Table 2 and Online Resources 1 and 2). The median activity of FVIII was higher than 150% 24 and 48 h after all evaluated IVMP pulses.

### Long-term influence of GCs on hemostasis during the entire therapy (between 1st, 6th and 12th IVMP pulses)

No statistically significant changes in hemostatic parameters were observed in long-term evaluation (Table 3). We observed increase of FII after 12th, FV after 6th, FVIII after 6th, PLT level after 6th and 12th IVMP pulse in comparison to the 1st pulse, but without statistical significance (after Bonferroni correction).

**Table 3:** Changes in coagulation parameters prior to selected intravenous methylprednisolone pulses

Coagulation parameter	Before 1st pulse	Before 6th pulse	Before 12th pulse
FII (Reference range 70–120%)	92.5 (84–105)	101.5 (86–113)	104 (93–117)^b^
FV (Reference range 70–120%)	93.5 (78–109)	106 (93–115)^a^	100 (94–112)
FVII (Reference range 70–120%)	92 (81–112)	96 (82–115)	105 (85–119)
FVIII (Reference range 70–150%)	140.2 (109.4–163)	154.9 (124.6–188.1)^b^	132.5 (107.9–184.7)
PT (Reference range 12–16 s)	15.1 (14.5–16.4)	15 (14.2–16)	15 (14.5–15.9)
INR (Reference range < 1.3)	0.97 (0.93–1.05)	0.96 (0.92–1.01)	0.98 (0.91–1.01)
aPTT (Reference range 25–37 s)	31 (29–33)	31 (29–33)	31 (28–34)
Fibrinogen (Reference range 200–400 mg/dl)	321.5 (255–345)	302 (255–353)	303 (261–371)
AT (Reference range 80–120%)	110.5 (104–115)	109.5 (102–113)	108 (101–115)
D-dimer (Reference range < 500 ng/dl)	311 (226–395)	336.5 (190–456)	290 (212–427)
PLT (Reference range 150–400R10^3^/µl)	233 (190–266)	241.5 (207–284)^a^	245 (201–289)^a^

Results are demonstrated as median values (lower quartile–upper quartile)

*FII* factor II, *FV* factor V, *FVII* factor VII, *FVIII* factor VIII, *PT* prothrombin time, *INR* international normalized ratio of prothrombin time, *aPTT* activated partial thromboplastin time, *AT* antithrombin, *PLT* platelets

After Bonferroni correction, results were claimed statistically significant with *p* value of < 0.0005

Statistical analysis was performed with paired *t* student test (parametric data) or with Wilcoxson test (non-parametric data)

^a^*p* < 0.05; ^b^*p* < 0.01; ^c^*p* < 0.0005; ^d^*p* < 0.00005; *p* values refer to comparisons with levels of coagulation parameters before the 1st pulse

### Patient with pulmonary embolism

Therapy was concluded in one patient after the 9th pulse due to pulmonary embolism. The patient was a 67-year-old, obese man (BMI 34.8 kg/m^2^) with well-controlled hypertension and a history of smoking. The patient was also being treated for chronic obstructive pulmonary disease. Moreover, an 8-h journey with immobilisation presented an additional risk factor. Detailed evaluation revealed correct coagulation parameters before therapy initiation (D-dimer, aPTT, PT, INR, fibrinogen, PLT, AT, F: II, V, VII, VIII within the normal range). We observed a gradual increase of FVIII activity during the 1st and 6th IVMP pulses, from 118 to 219%, together with a shortening of aPTT from 35 to 29 s. Other coagulation parameters mentioned above remained within the reference ranges. Before administering the 10th pulse, which was consequentially cancelled, the patient had been complaining of increasing dyspnoea on exertion. The level of D-dimers increased to 7983 ng/dl and the diagnosis of thrombosis of the right posterior tibial vein, followed by PE, was made. Anticoagulation therapy with enoxaparin was initiated, followed by acenocoumarol treatment. The level of CRP at the time of thrombosis was 1.4 mg/l (reference range 0–10 mg/l). The patient was excluded from further participation in steroid therapy. The cumulative dose of IVMP received by the patient was 3.75 g.

### Comparison of independent groups (patients with initially increased/reduced selected markers versus without increased/reduced selected markers), according to basal characteristics

According to the basal characteristics of patients, there were not any statistically significant differences observed in clinical data (such as age, sex, body mass index, smoking, duration time of GO, presence of hypertension, basal markers of thyroid function) between independent groups (patients with initially increased/reduced selected markers versus without increased/reduced selected markers) (See Online Resource 3).

## Discussion

IVMP pulses therapy as the first-line treatment for moderate-to-severe and active GO is more effective and has less side effects than orally administered GCs therapy [12–14]. Patients unresponsive after the 6th pulse with IVMP still have a significant possibility of improvement later [20]. However, there are some reports of fatal adverse effects associated with this treatment even after smaller cumulative IVMP doses. In the Bartalena et al. study, there was a case of death due to myocardial infarction 1 week after the 6th infusion of once-weekly treatment with 0.25 g MP/one pulse with cumulative dose reaching 1.5 g [19]. Adverse effects of IVMP pulse therapy are probably partly due to a hypercoagulable state.

This is the first prospective study with such a complex analysis of the influence of the high-doses IVMP pulse therapy on hemostatic process after selected (1st, 6th and 12th) IVMP pulses together with long-term evaluation. We found a significant increase of activity of FVIII 24 and 48 h after every IVMP pulse with simultaneous shortening of aPTT 24 h after each IVMP pulse. Increased FVIII activity with a shortening of aPTT indicates activation of the intrinsic system after every single IVMP administration regardless of dose (0.5 and 0.25 g), especially during the first 24 h. The median activity of FVIII was higher than 150% after 24 and 48 h after all IVMP pulses. No statistically significant changes in hemostatic parameters were observed in long-term evaluation. The elevated plasma activity of FVIII is a crucial, predominant independent and dose-dependent risk factor for VTE and recurrent VTE [21]. Plasma activity of FVIII above 150% is associated with adjusted thrombosis risk about 4.8 [22].

Numerous alterations of coagulation and fibrinolysis parameters have been described among patients with endogenous Cushing’s syndrome (CS) [1–6] and those treated with GCs [6–8].

Patients with CS present higher levels of: FII, FV, FVIII, FIX, FXI, FXII, FVIII, and von Willebrand factor (vWF), as well as increased levels of fibrinolytic inhibitors, e.g., plasminogen activator inhibitor type 1 [1–4, 23]. Impact on hypercoagulability in CS depends on the degree of hypercortisolism. In Swiatkowska et al. study, activity of FVIII was higher only in patients with overt CS, not with subclinical CS; however, vWF was increased in both groups [23]. The hypercoagulable state and impairment of fibrinolytic capacity in CS are associated with an increased risk of VTE and PE [5, 9]. The most likely underlying mechanism for this phenomenon is cortisol-induced upregulation of mRNA transcription of various coagulation factors [24]. Anticoagulation prophylaxis reduces thromboembolic complications in Cushing’s disease [4]. According to the Endocrine Society’s recommendations, patients with CS should be evaluated for risk factors of venous thrombosis and in case of surgery; perioperative prophylaxis for VTE is advised [25].

Prolonged GCs therapy with higher doses can lead to a hypercoagulable state and VTE [10, 11]. In a prospective, randomized study Jilma et al. demonstrated in 9 healthy men the short-term influence of cumulative high doses of intravenous dexamethasone (1 mg/kg) on the increase of the plasma levels of vWF and sP-selectin by about 15–20%, which was not observed after lower doses of dexamethasone (0.04 mg/kg) and in the placebo group [8]. This study indicated that high dose dexamethasone up-regulates vWF transcription and does not regulate secretion of vWF. In a randomized, placebo-controlled study performed by Brotman et al. 24 healthy men received 3 mg of dexamethasone twice daily versus placebo for 5 days [7]. There was a significant increase in FVII, FVIII and FXI found in the dexamethasone group, while fibrinolytic activity was not decreased (plasminogen activator inhibitor type 1 levels were not changed significantly) and D-dimer levels were not changed. Frank et al. studied the impact of high-dose intravenous MP (500 mg per day for 5 days) simultaneously with anticoagulation prophylaxis (2500 U dalteparin) in 10 patients with multiple sclerosis [11]. There was a moderate increase in FVIII and vWF antigen levels observed, albeit in 9 out of 10 patients FVIII activity remained within the range of normal control subjects (< 150%).

It should be noticed that the basic activity of FVIII (before administration of IVMP) was above reference range (higher than 150%) in 38% of our patients. Hyperthyroidism is associated with increased activity of FVIII, achievement of euthyroidism in hyperthyroid patients leads to normalization of the previously elevated FVIII [26]. We have to underline that all of our patients were in a euthyroid state during the study. We consider that increased activity of FVIII at this point of evaluation was not associated with successfully treated hyperthyroidism. In some studies, elevated levels of FVIII (> 150%) were observed in controls with a prevalence of 2–38% [21, 27, 28]. In Kraaijenhagen et al. study, FVIII activity was higher than 175 in 10% of control group [21]. Plasma activity of FVIII increases with age; this could partially explain the higher basic activity of this factor in our study than in other studies. We cannot exclude the influence of obesity, notwithstanding. We noticed a higher prevalence, without statistical significance, of increased basic FVIII activity in obese versus non-obese patients.

Additionally, we observed that fibrinogen decreased 48 h after each pulse. The potential mechanism can be associated with reduced synthesis or secretion after IVMP. D-dimer levels has not changed during therapy (except one patient with PE) that indicates against activation of consumption. Fibrinogen level and CRP were within normal ranges in all patients before initiation of therapy, indicating the lack of inflammation.

Contrary to previous studies, there was a decrease of FVII, a factor typically involved in the activation of the extrinsic pathway, 24 h after each IVMP pulse [7]. This trend may be a compensatory mechanism in patients with an activated intrinsic pathway and requires further investigation.

PE was diagnosed in one patient after the 9th IVMP pulse. This patient was obese, with hypertension, chronic obstructive pulmonary disease and a long history of smoking. An additional risk factor (an 8-h journey with immobilisation) could be provocative for the resulting thromboembolism. However, we cannot exclude IVMP as the additional factor for the hypercoagulable state. Interestingly, the activity of FVIII in this patient before therapy was within the normal range, but during long-term evaluation (between the 1st and 6th IVMP pulses) the gradual increase from 118 to 219% was observed along with a shortening of aPTT from 35 to 29 s. Therefore, we suggest that, in some patients with additional risk factors (immobilisation, obesity, and smoking) together with the IVMP therapy anticoagulation prophylaxis should be taken into consideration.

The main question that comes to us is if changes in hemostatic parameters during IVMP therapy in weekly scheduled pulses are associated with higher risk of VTE. According to our results, an influence on clothing factors occurred after each pulse but is transient. The opposite situation is observed in patients with CS, in whom hypercoagulability remains during the whole period of hypercortisolemia [23, 29]. GCs exert most of their effects through the classical genomic mechanism. Anti-inflammatory effects of GCs are mostly due to inhibition of transcription (transrepression), whereas the activation of transcription (transactivation) by the GCs receptor accounts for the majority of side effects [30]. Non-genomic activity might become relatively more important in mediating the therapeutic effects of high-dose pulsed GCs and might produce lesser side effects [30, 31]. Transient impact of hypercoagulability after IVMP weekly scheduled pulses (in doses ≤ 0.5 g) is probably safe in most of the patients without additional risk factors for VTE. Therapy with IVMP in consecutive days, with higher single pulses (1.0 g/day) [32] and higher cumulative doses (> 8.0 g) [32] is associated with higher risk of adverse effects. We do not know if the balance between genomic and non-genomic mechanism plays a rule in impact of this effect.

The disadvantage of this trial is the small sample size of the study group. However, results were statistically significant and the same after each analysed pulse that confirmed stable trend (Table 2 and Online Resources 1 and 2). Analysis in a larger group would give additional information according to long-term influence of IVMP on hemostatic factors that were higher after therapy but without a significant difference (FII, FV, FVIII). We did not analyse the influence of IVMP on vWF, markers of fibrinolysis and parameters of thrombin generation. Some of them were analysed in previous studies (vWF in Jilma et al. study, PAI-1 in Brotman et al. study) [7, 8], however, analysing these parameters in the bigger group could be beneficial in understanding the whole hemostatic process in patients treated with IVMP.

To conclude, the impact of treatment with IVMP on hemostatic process in patients with GO is associated with the increase of FVIII that occurs after each pulse and is transient. In patients with additional risk factors of VTE, anticoagulation prophylaxis should be considered.

## Electronic supplementary material

Below is the link to the electronic supplementary material.

Supplementary material 1 (PDF 78 kb)

Supplementary material 2 (PDF 155 kb)

Supplementary material 3 (PDF 130 kb)

## Abbreviations

aPTT: Activated partial thromboplastin time
AT: Antithrombin
CRP: C-reactive protein
CS: Cushing’s syndrome
GCs: Glucocorticosteroids
GO: Graves’ orbitopathy
EUGOGO: European Group On Graves’ Orbitopathy
FII: Factor II
FV: Factor V
FVII: Factor VII
FVIII: Factor VIII
FT4: Free thyroxine
INR: International normalized ratio of prothrombin time
IVMP: Intravenous methylprednisolone
PE: Pulmonary embolism
PT: Prothrombin time
PLT: Platelet count
VTE: Venous thromboembolic events
TSH: Thyroid stimulating hormone
