# Supplementary material for: High-dose intravenous methylprednisolone therapy in patients with Graves’ orbitopathy is associated with the increased activity of factor VIII
Source: J Endocrinol Invest. 2018 Jun 9;42(2):217–25. doi: 10.1007/s40618-018-0907-z (PMC6394431; doi:10.1007/s40618-018-0907-z)
Supplement: Supplementary file 1 — Supplementary material 1 (PDF 78 kb) [file 40618_2018_907_MOESM1_ESM.pdf]

Journal of Endocrinological Investigation, “High-dose intravenous methylprednisolone therapy in patients with Graves’ orbitopathy is associated with the increased activity of factor VIII.”
Authors: Piotr Miśkiewicz[1], Justyna Milczarek-Banach[1], Beata Rutkowska - Hinc[1], Agnieszka Kondracka[1], Tomasz Bednarczuk[1]
[1]Department of Internal Medicine and Endocrinology, Medical University of Warsaw, Banacha 1a, 02-097 Warsaw, Poland
Correspondence: piotr.miskiewicz@wum.edu.pl

Online Resource 1. Changes in coagulation parameters during 6th intravenous methylprednisolone pulse (0.5 g).

Coagulation parameter	Before pulse	24h after pulse	48h after pulse
**FII** (reference range 70-120%)	101,5 (86-113)	103,5 (97-118)	101 (84-113)
**FV** (reference range 70-120%)	106 (93-115)	**118 (106-130)**[d]	103 (89-116)
**FVII** (reference range 70-120%)	96 (82-115)	**84 (72-99)**[d]	90 (80-109)
**FVIII** (reference range 70-150%)	154,9 (124,6-188,1)	**190,3 (154,5-226,2)**[d]	**180,2 (137,7-234,8)**[d]
**PT** (reference range 12-16 s)	14,95 (14,2-16)	**15,05 (14,8-15,95)**[c]	15,4 (14,8-16)[b]
**INR** (reference range <1.3)	0,96 (0,92-1,01)	0,99 (0,95-1,05)[b]	0,99 (0,94-1,06)[b]
**aPTT** (reference range 25-37 s)	31 (29-33)	**29 (27-31)**[d]	28 (27-31)**
**Fibrinogen** (reference range 200-400 mg/dl)	302 (255-353)	327 (277,5-353)	**263 (230-320)**[d]
**AT** (reference range 80-120%)	109,5 (102-113)	114 (107-124)[a]	114 (105-116)[a]
**D-dimer** (reference range <500 ng/dl)	336,5 (190-456)	284 (162-436)[b]	280 (180-349)[b]
**PLT** (reference range 150-400x10^3/µl)	241,5 (207-284)	267 (221-316)[b]	239 (187-296)

FII – factor II, FV – factor V, FVII – factor VII, FVIII – factor VIII, PT – prothrombin time, INR – international normalized ratio of prothrombin time, aPTT – activated partial thromboplastin time, AT – antithrombin, PLT – platelets.
Results are demonstrated as median values (lower quartile – upper quartile).
[a] $p<0.05$; [b] $p<0.01$; [c] $p<0.0005$; [d] $p<0.00005$, p values refer to comparisons with levels of coagulation parameters before the 6th pulse.
After Bonferroni correction, results were claimed statistically significant with p value of <0.0005 (bolded).
Statistical analysis was performed with paired t-student test (parametric data) or with Wilcoxson test (non-parametric data).
