# Supplementary material for: High-dose intravenous methylprednisolone therapy in patients with Graves’ orbitopathy is associated with the increased activity of factor VIII
Source: J Endocrinol Invest. 2018 Jun 9;42(2):217–25. doi: 10.1007/s40618-018-0907-z (PMC6394431; doi:10.1007/s40618-018-0907-z)
Supplement: Supplementary file 2 — Supplementary material 2 (PDF 155 kb) [file 40618_2018_907_MOESM2_ESM.pdf]

Journal of Endocrinological Investigation, "High-dose intravenous methylprednisolone therapy in patients with Graves' orbitopathy is associated with the increased activity of factor VIII."
Authors: Piotr Miśkiewicz[1], Justyna Milczarek-Banach[1], Beata Rutkowska - Hinc[1], Agnieszka Kondracka[1], Tomasz Bednarczuk[1]
[1]Department of Internal Medicine and Endocrinology, Medical University of Warsaw, Banacha 1a, 02-097 Warsaw, Poland
Correspondence: piotr.miskiewicz@wum.edu.pl

Online Resource 2. Changes in coagulation parameters during 12th intravenous methylprednisolone pulse.

Coagulation parameter	Before pulse	24h after pulse	48h after pulse
**FII** (reference range 70-120%)	104 (93-117)	106 (92-116)	103,5 (92,5-114,5)
**FV** (reference range 70-120%)	106 (93-115)	109 (99-130)^{b}	101,5 (90,5-117)
**FVII** (reference range 70-120%)	105 (85-119)	**85 (78-96)**^{c}	95,5 (79-104)^{b}
**FVIII** (reference range 70-150%)	132,5 (107,9-184,7)	**181,4 (142,3-216,8)**^{d}	**159,7 (127,9-207)**^{d}
**PT** (reference range 12-16 s)	15 (14,5-15,9)	15,05 (14,8-15,95)	15,25 (14,45-16,05)
**INR** (reference range <1.3)	0,98 (0,91-1,01)	0,99 (0,95-1,05)	0,98 (0,94-1,055)
**aPTT** (reference range 25-37 s)	31 (28-34)	**28 (26-30)**^{d}	**29 (26,15-31)**^{c}
**Fibrinogen** (reference range 200-400 mg/dl)	303 (261-371)	327 (277,5-353)	**259 (220-323)**^{d}
**AT** (reference range 80-120%)	108 (101-115)	**113,5 (107,5-118)**^{c}	113 (101-122)^{a}
**D-dimer** (reference range <500 ng/dl)	290 (212-427)	272 (207,5-396,5)	265,5 (200-387,5)^{a}
**PLT** (reference range 150-400x10^3/µl)	245 (201-289)	**267 (221-316)**^{c}	247 (208-291)

FII – factor II, FV – factor V, FVII – factor VII, FVIII – factor VIII, PT – prothrombin time, INR – international normalized ratio of prothrombin time, aPTT – activated partial thromboplastin time, AT – antithrombin, PLT – platelets.
Results are demonstrated as median values (lower quartile – upper quartile).
^{a} p<0.05; ^{b} p<0.01; ^{c} p<0.0005; ^{d} p<0.00005, p values refer to comparisons with levels of coagulation parameters before the12^{th} pulse.
After Bonferroni correction, results were claimed statistically significant with p value of <0.0005 (bolded).
Statistical analysis was performed with paired t-student test (parametric data) or with Wilcoxson test (non-parametric data).
