# Supplementary material for: High-dose intravenous methylprednisolone therapy in patients with Graves’ orbitopathy is associated with the increased activity of factor VIII
Source: J Endocrinol Invest. 2018 Jun 9;42(2):217–25. doi: 10.1007/s40618-018-0907-z (PMC6394431; doi:10.1007/s40618-018-0907-z)
Supplement: Supplementary file 3 — Supplementary material 3 (PDF 130 kb) [file 40618_2018_907_MOESM3_ESM.pdf]

Journal of Endocrinological Investigation, “High-dose intravenous methylprednisolone therapy in patients with Graves’ orbitopathy is associated with the increased activity of factor VIII.”
Authors: Piotr Miśkiewicz[1], Justyna Milczarek-Banach[1], Beata Rutkowska - Hinc[1], Agnieszka Kondracka[1], Tomasz Bednarczuk[1]
[1]Department of Internal Medicine and Endocrinology, Medical University of Warsaw, Banacha 1a, 02-097 Warsaw, Poland
Correspondence: piotr.miskiewicz@wum.edu.pl

Online Resource 3. Comparison of groups of patients with initially increased/decreased selected coagulation parameters according to basal characteristics.

**Clinical data**	***FVIII***			***FVII***			***Fibrinogen***		
	**>150% (n=10)**	**≤150% (n=16)**	**p**	**>120% (n=4)**	**≤120% (n=22)**	**p**	**>400 mg/dl (n=3)**	**≤400 mg/dl (n=23)**	**p**
Female	7	8	0.32	3	12	0.45	2	13	0.74
Duration of GO (weeks)	76	30	0.03	80	39	0.34	55	41	0.83
Smoking (current or former)	8	14	0.61	4	18	0.35	3	19	0.43
BMI ≥ 30 kg/m^2	3	2	0.27	1	4	0.75	2	3	0.03
Hypertension	6	5	0.15	2	9	0.74	3	8	0.03
Age (years)	52	54	0.62	58	50	0.23	61	50	0.06
Baseline TSH (μIU/ml)	1.61	1.37	0.23	1.34	1.47	0.70	1.47	1.47	0.94
Baseline FT4 (pmol/l)	16	15	0.96	16	16	0.70	17	12	0.94

FVIII – factor VIII, FVII – factor VII, n – number of subjects, GO – Graves’ orbitopathy, BMI – body mass index, TSH – thyroid stimulating hormone, FT4 – free thyroxine

Results were demonstrated as number of subjects for categorical data or median values for continuous data.
After Bonferroni correction, results were claimed statistically significant with p value of <0.0005.
Statistical analysis was performed with chi-squared method for categorical variables and Mann-Whitney U test for continuous variables.
